# Activation of protein kinase A (PKA) by 8-Cl-cAMP as a novel approach for antileukaemic therapy

**DOI:** 10.1038/sj.bjc.6601909

**Published:** 2004-06-08

**Authors:** E M Weissinger, K Oettrich, C Evans, H-G Genieser, F Schwede, M Dangers, E Dammann, H-J Kolb, H Mischak, A Ganser, W Kolch

**Affiliations:** 1 Medizinische Hochschule Hannover (MHH), Department of Hematology and Oncology, Hannover, Germany; 2 Mosaiques diagnostics and therapeutics AG, Hannover, Germany; 3 Klinikum Großhadern, Clinical Cooperative Group Hematopoietic Cell Transplantation, Munich, Germany; 4 Leukaemia Research Fund Cellular Development Unit, UMIST, Manchester UK; 5 LRF Proteomics Facility, UMIST, UK; 6 Biolog Life Science Institute, Bremen, Germany; 7 MHH, Department of Nephrology, Hannover Germany; 8 Beatson Institute for Cancer Research, Signalling and Proteomics Group, Garscube Estate, Glasgow, UK; 9 Institute for Biomedical and Life Sciences, University of Glasgow, Glasgow, UK

**Keywords:** leukaemia, purging, protein kinase A, 8-Cl-cAMP

## Abstract

Activation of PKA by cAMP agonists, such as 8-Cl-cAMP activation, selectively causes rapid apoptosis in v-abl transformed fibroblasts by inhibiting the Raf-1 kinase. Here we investigated whether 8-Cl-cAMP is useful for the treatment of chronic myelogenous leukaemia (CML), which is hallmarked by the expression of the p210^*bcr/abl*^ oncogene. Autologous bone marrow transplantation is a feasible alternative for patients with no suitable donor, but hampered by the risk of relapse due to the persistence of leukaemia cells in the transplant. To study the effects of 8-Cl-cAMP on primary leukaemic cells, bone marrow cells (BMCs) from eight CML patients (one at diagnosis, three in chronic and four in accelerated phase) were treated. *Ex vivo* treatment of BMCs obtained in chronic phase of CML with 100 *μ*M 8-Cl-cAMP for 24–48 h led to the selective purging of Philadelphia Chromosome (Ph1 chromosome) without toxic side effects on BMCs from healthy donors as measured by colony-forming unit (CFU) assays. BMCs from patients in accelerated phase showed selective, but incomplete elimination of Ph1 chromosome positive colony forming cells. The mechanism of 8-Cl-cAMP was investigated in FDCP-mix cells transformed by p210^*bcr/abl*^, a cell culture model for CML. The results showed that 8-Cl-cAMP reduced DNA synthesis and viability independent of Raf inhibition as Raf inhibitors had no effect. MEK inhibitors interfered with DNA synthesis, but not with viability. In summary, our results indicate that 8-Cl-cAMP could be useful to purge malignant cells from the bone marrow of patients with CML and certain other forms of leukaemias.

The activation of signalling pathways due to the constitutive expression of the Bcr-Abl oncogene plays a major role in the pathogenesis of some leukaemias, in particular chronic myelogenous leukaemia (CML). CML is the major adulthood leukaemia characterised by the Philadelphia chromosome (Ph1 chromosome, t:9/22), a chromosomal translocation where *bcr* sequences from chromosome 22 are juxtaposed to *c*-*abl* on chromosome 9 leading to the expression of an atypical fusion-protein p210^*bcr-abl*^ (Butturini *et al*, 1996). In its chronic phase CML is hallmarked by abnormally sustained cell survival rather than excessive proliferation and is relatively well controlled by cytoreductive chemotherapy. However, the chronic phase inevitably turns into an acute phase of blast crisis where leukaemic blast cells proliferate rapidly and aggressively with fatal consequences.

p210^*bcr-abl*^ is a constitutively activated tyrosine kinase that activates numerous cellular signalling pathways including the Raf–MEK–ERK pathway, which is critical for malignant transformation. A tyrosine kinase inhibitor for p210^*bcr-abl*^, CGP-57148 now called STI571, has sparked great interest as it dramatically increased the number of patients achieving complete remission. However, almost half of the patients treated in the chronic phase remain Ph1 chromosome positive with the inherent risk of relapse. Indeed, many patients treated with STI571 in the acute phase relapse rapidly (La Rosee *et al*, 2002). This probably relates to the fact that STI571 is inhibiting proliferation rather than eliminating the leukaemic cells (Beran *et al*, 1998; La Rosee *et al*, 2002). Despite the addition of STI571 to the clinical arsenal bone marrow and peripheral blood stem cell transplantation (PBSCT) remains a mainstay of therapy. For autologous PBSCT stem cells are harvested from peripheral blood after stimulation with G-CSF at the time of clinical remission. A proportion of patients responds at least initially to autologous PBSCT (Reiffers *et al*, 1994). Regardless whether PBSCT or autologous bone marrow transplantation is used, it is crucial to eliminate leukaemic cells from the transplant in order to avoid the transfer of leukaemic cells back to the patient.

For CML the logical target that distinguishes normal cells from leukaemic cells is p210^*bcr-abl*^. This fusion protein aberrantly activates a number of signalling pathways that act in concert to transform cells. Thus, these pathways emanating from p210^*bcr-abl*^ are all potential targets for therapeutic intervention. Prominent targets are the Raf-1 kinase and the c-Myc transcription factor. p210^*bcr-abl*^ induces c-Myc expression in haematopoietic cells (Sawyers *et al*, 1992; Sawyers, 1993; Weissinger *et al*, 1993). The deregulation of c-Myc expression has been shown to be required for transformation by oncogenic abl genes (Sawyers, 1993; Weissinger *et al*, 1993). p210^*bcr-abl*^ also activates Raf-1 and the MEK–ERK pathway. The canonical Raf-MEK–ERK pathway is often perceived as a linear signalling module that mediates cell proliferation, transformation and survival (Weissinger *et al*, 1997). We have shown previously that the activation of the cAMP dependent protein kinase A (PKA) with synthetic agonist drugs such as 8-Chloro-cyclic Adenosine Monophosphate (8-Cl-cAMP) results in the inhibition of Raf-1 kinase activity and rapid apoptosis induction in v-abl transformed fibroblasts. Apoptosis occurred despite a high constitutive activity of ERK suggesting that Raf-1 uses a different pathway to ensure cell viability (Weissinger *et al*, 1997).

These observations led us to explore the use of 8-Cl-cAMP for the treatment of CML bone marrow cells for *ex vivo* for the purging of leukaemic bone marrow or for the treatment of leukaemic patients. 8-Cl-cAMP is one of the most stable compounds that activate PKA (Schwede *et al*, 2000) and can be manufactured in quantities and quality sufficient for clinical use. In fact, the antitumour activity of 8-Cl-cAMP has been under study for a number of years, including clinical studies for the treatment of tumours (Cho-Chung *et al*, 1995; Tortora *et al*, 1995; Propper *et al*, 1999). To date, mainly solid tumours like breast carcinomas were studied. In this report we present data indicating that treatment with 8-Cl-cAMP can provide an effective method for purging bone marrow prior to autologous transplantation, targeting specifically the p210^bcr/abl^ transformed cells.

## PATIENTS AND METHODS

### Patients

The studies were approved by the institutional ethics committees of Munich and Hannover. Bone marrow from healthy donors, eight patients with CML (five males and three females age range: 25–49) in haematological chronic phase or at more advanced stages was obtained after informed consent.

### Synthesis of 8-Cl-cAMP

Preparation of 8-Cl-cAMP was performed as described with minor modifications (Brentnall and Hutchinson, 1972; Schwede *et al*, 2000). Briefly, 30 g (85.47 mmol) cAMP, sodium salt, were suspended in 2000 ml DMF and reacted with 87 g (171 mmol) tetrabutylammonium iodotetrachloride for 18 h. The reaction mixture was poured into 3000 ml water and extracted with chloroform (3 × 250 ml) to remove excess of reagent. The resulting solution was concentrated under reduced pressure. 8-Cl-cAMP was purified and isolated by means of column liquid chromatography using silica-base reversed phase material (Merck, Germany, LiChroprep® Rp-18) (Cummings *et al*, 1994). The product containing fractions were collected and evaporated to produce 13.95 g (36.18 mmol) 8-Cl-cAMP, sodium salt, with a purity of >99% (yield: 42.3%).

### Cell lines

FDCP-mix cells expressing temperature-sensitive p210^*bcr/abl*^ (Pierce *et al*, 1998) were cultured in Fisher's medium, 20% v v^−1^ horse serum and 5% v v^−1^ murine IL-3 conditioned medium (CM) from the X63Ag8-653 myeloma cell line as a source of murine IL-3 (Karasuyama and Melchers, 1988).

### Long-term culture and colony-forming units (CFU)

**Figure 1:**
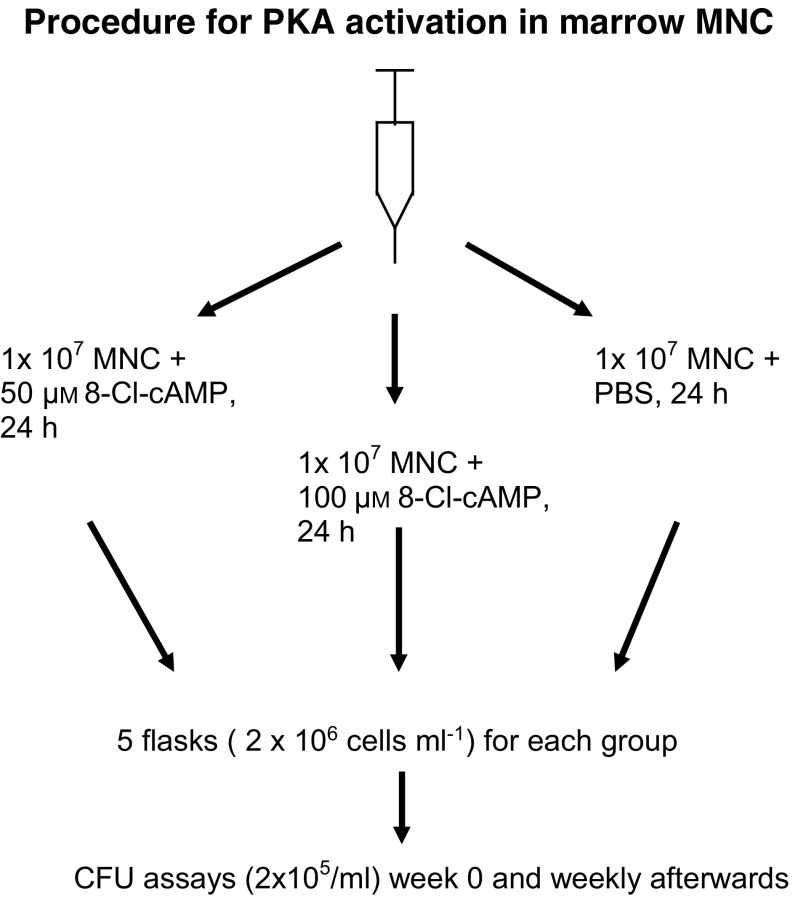
Procedure for PKA activation in bone marrow mononuclear cells (MNC). Marrow MNC were harvested, washed and resuspended in LTC medium. Separate flasks were generated for long-term culture and 8-Cl-cAMP was added as indicated. After 24 h cells were washed, and expanded in long-term cultures as described. CFU assays were setup weekly with the nonadherent cells (NADC). At the end of the long-term culture, adherent cells (ADC) and nonadherent cells (NADC) were harvested and analysed in separate CFU-assays.

The cells were resuspended in LTC-medium supplemented with 12.5% horse serum (Hyclone, Munich, Germany) and 10^−5^ M hydrocortisone at a density of 2 × 10^6^ cells ml^−1^ and cultured for 4–6 weeks on autologous feeder layers (LTC-medium, Iscoves Mod. Dulbecco's medium 340 mOsm, Gibco BRL, Bethesda, USA). Cells were cultured in duplicates and incubated with medium alone, 50 or 100 *μ*M 8-Cl-cAMP for 24 h (summarised in Figure 1

). Subsequently, cells were washed and resuspended in LTC medium. Nonadherent cells (NADC) were harvested in weekly intervals and resuspended in MethoCult GF H4434 (Stem Cell Technology, Munich, Germany). Colonies were counted after 7 days and evaluated according to standard clinical haematological technique.

### Nonspecific toxicity testing of 8-Cl-cAMP

The nonspecific toxicity of 8-Cl-cAMP on bone marrow cells was tested using marrow mononuclear cells (MNC) from healthy donors as outlined in Figure 1. The MNCs were cultured at a density of 2 × 10^6^ cells ml^−1^ in LTC medium as described above in the presence or absence of 8-Cl-cAMP for 24 h. Subsequently, the cells were washed and grown in long-term culture medium. In total, 2 × 10^5^ cells ml^−1^ were used for CFU assays. At the end of the long-term culture (4–6 weeks), the adherent cells (ADC) as well as the NADC were harvested and analysed in the same manner.

### Cytogenetic analyses

After completion of the CFU assays, colonies were picked and analysed for Ph1 chromosome positive colonies. Cytogenetic analyses were essentially performed as described (Dube *et al*, 1981). Briefly, cell division was arrested in metaphase by the addition of 100 *μ*l of a colchicine solution (1 *μ*g ml^−1^ in *α*-minimal essential medium) to 1 ml of cell suspension for 12 h. The cells were subsequently transferred to poly-L-lysine-coated slides and incubated in 0.2 ml of 0.075 mol l^−1^ KCl at room temperature for 10 min. Cells were fixed by gently dropping 100 *μ*l cold methanol on the slide. The excess of the fixative was removed with absorbent paper and slides were dried on a hot plate at 55°C. After repeated cold fixation for 15 min, the cells were used for banding and cytogenetic analyses.

### Growth curves

8-Cl-cAMP was added to the cultures as indicated on day 0 and was not replenished during the culture. FDCP-mix cells were plated in 24-well plates at a density of 2 × 10^5^ cells ml^−1^. The MEK inhibitors (U0126, PD98059) and raf kinase inhibitors, raf kinase inhibitor 1 (Raf KI) and ZM 336372, were purchased from Calbiochem, UK. Survival and proliferation assays were performed as previously described (Pierce *et al*, 1998).

### Viability assay

FDCP-mix cells were washed and resuspended (4 × 10^5^ cells ml^−1^) in Fisher's medium supplemented with 20% (v v^−1^) HS. The cells were treated with 8-Cl-cAMP (100 *μ*M) in the absence of murine IL-3 (0.5ng ml^−1^). Samples were taken after 24, 48 and 72 h in culture. Cell viability was analysed by flow cytometry using the annexin V-FITC, propidium iodide (PI) based assay (R&D Systems, Oxford, UK) as previously described (Francis *et al*, 2000). Samples were analysed using a FacsVantage flow cytometer (Becton Dickinson Co., Mountain View, CA, USA). Viable cells (unstained), early apoptotic cells (Annexin V positive) and late apoptotic and/or necrotic cells were analysed. Results are shown±s.e.m. (*n*=3).

## RESULTS

### PKA-activation resulted in a transient growth inhibition of human bone marrow cells

The nonspecific toxicity of 8-Cl-cAMP was tested on human bone marrow cells (BMC) of healthy donors (Table 1

**Table 1:** Colony formation assays and cytogenetic analyses on normal volunteer marrow MNC

**Cultures**		**Granulocytes (G)**	**Eosinophiles (Eo)**	**Macrophages (M)**	**Erythrocytes (E)**	**Mixed colonies (GM)**	**Blasts**	**Total no.**
Week 0 control		49	2	61	128	12	7	259
8-Cl-cAMP (100 *μ*m)		27	4	18	100	7	9	145
Week 5 control	ADC	57	2	16	6	2	5	88
8-Cl-cAMP(100 *μ*m)	ADC	70	2	10	30	14	22	148

Data obtained after treatment of normal marrow MNC with 8-Cl-cAMP are summarised. The cells were resuspended at 2.5 × 10^5^ cells ml^−1^ as described in Figure 1. The effects of treatment with 8-Cl-cAMP (one single exposure of the cells for 24 h) on colony formation was observed at week 0 and after week 5.2 × 10^5^ cells ml^−1^ were plated in soft agar for colony formation in week 1 or after long-term culture. The nonadherent cell fraction did not yield significant amounts of colonies with or without 8-Cl-cAMP treatment after 5 weeks, thus only the adherent cells (ADC) are shown. Colonies were judged by their typical histological appearance according to haematological criteria: G=granulocyte; Eo=eosinophiles; M=macrophages; E=erythrocytes; GM=mixed colonies, Granulocytes and macrophages.

). In total, 15 flasks of cells per donor were set up for long-term cultures after treatment with 8-Cl-cAMP, five for each condition (control, 50 *μ*M and 100 *μ*M 8-Cl-cAMP; Figure 1). BMCs taken at week 0 immediately after treatment with 8-Cl-cAMP showed a reduction of cell numbers and a concomitant reduction of colonies arising in the CFU assays. However, at all subsequent timepoints, ranging from 1 to 5 weeks, comparable numbers of CFU-initiating cells were obtained from treated and untreated BMC cultures. This is summarised in Table 1 showing CFUs obtained from week 5 cultures as example. Thus, 8-Cl-cAMP only caused a transient impairment of CFU capacity in BMCs from healthy donors that was readily reversed at longer time points. This is consistent with our earlier observations (Weissinger *et al*, 1997) that untransformed cells only respond to PKA activation with an initial, transient inhibition of proliferation.

### Ph1 chromosome positive colony forming cells are selectively eliminated by 8-Cl-cAMP

Since no severe nonspecific toxicity was observed that would prohibit treatment with 8-Cl-cAMP, bone marrow MNCs of eight patients with CML were treated in the same manner (Table 2

**Table 2:** Percentage Philadelphia chromosome positive colonies (CFU-GM) in patients with CML in the presence or absence of 8Cl-cAMP

	**Week 0**			**Week 5**		
**Patient ID**	**Control**	**8Cl-cAMP 50 *μ*m**	**8Cl-cAMP 100 *μ*m**	**Control**	**8Cl-cAMP 50 *μ*m**	**8Cl-cAMP 100 *μ*m**
1216 (cp)	100 (2/2)	100 (20/20)	100 (28/28)	ND	14 (3/21)	0 (0/15)
1206 (cp)	100 (12/12)	ND	70 (12/17)	83 (30/36)	ND	0 (0/15)
1726 (cp)	100 (62/62)	ND	100 (25/25)	83 (26/29)	ND	0 (0/37)
166 (D)	100 (6/6)	100 (15/15)	ND	100 (20/20)	46 (7/15)	ND
A97/3 (ap)	100 (21/21)	ND	100 (30/30)	100 (5/5)	ND	83 (5/6)
371 (ap)	81 (27/33)	ND	92 (24/26)	81 (27/33)	ND	33 (1/3)
361 (ap)	100 (30/30)	ND	100 (19/19)	100 (30/30)	ND	50 (2/4)
341 (ap)	100 (40/40)	ND	96 (26/27)	100 (34/34)	ND	62 (5/8)

The Ph1 chromosome positive colonies (in %) arising from patient bone marrow cells in percent are summarised, the actual number of colonies is given in parenthesis (Ph1 chromosome+/total number). MNC were incubated for 24 h without or with 50 or 100 *μ*m of 8-Cl-cAMP as indicated, washed and resuspended in long-term culture medium (Dexter). CFU-assays were setup in week 0 and weekly thereafter. CFU-GM of the adherent fraction of the cells (ADC) is shown, since there were only few colonies in the non-adherent fraction at this time. Ph1 chromosome status was determined by cytogenetic analysis. cp=chronic phase; D=diagnosis; ap=accelerated phase; ND=not determined.

). Three patients were in the chronic phase of CML undergoing cytoreductive treatment, one patient was at diagnosis and four were in the accelerated phase or blast crisis. In all cases, treatment with 8-Cl-cAMP substantially reduced the number of Ph1 chromosome positive colonies. The incubation with 50 *μ*M 8-Cl-cAMP resulted in a reduction of Ph1 chromosome positive colonies to 14 and 46%, respectively, but never led to a complete elimination of the Ph1 chromosome positive progenitor cells. Therefore, 100 *μ*M 8-Cl-cAMP was used in further experiments.

In CFU assays prepared from cultures at week 0 all colonies were Ph1 chromosome positive, and no difference was observed between the treated and the untreated cells. However, treatment with 8-Cl-cAMP resulted in a significant reduction of Ph1 chromosome positive CFUs prepared after 5 weeks of culture depending on the stage of disease. In three patients undergoing cytoreductive therapy, the treatment with 100 *μ*M 8-Cl-cAMP led to a complete loss of Ph1 chromosome positive colonies after 5 weeks of culture. In the absence of 8-Cl-cAMP only a small reduction of Ph1 chromosome positive colonies was observed after 5 weeks of culture, with more than 80% of the colonies remaining Ph1 chromosome positive. Cells from one untreated patient and four patients in advanced stages of CML exhibited a reduction of cells with a significant loss of Ph1 chromosome positive colonies ranging from 83 to 33%. Interestingly, after treatment with 8-Cl-cAMP Ph1 chromosome negative colonies appeared in CFU assays prepared from patients in the accelerated phase of CML, whereas in the untreated controls 100% of the colonies were Ph1 chromosome positive.

### Studies to investigate the molecular mechanism of 8-Cl-cAMP

Given the encouraging results obtained with 8-Cl-cAMP in clinical samples and patients we investigated the molecular basis of the activity of 8-Cl-cAMP in particular with regard to its effects on cell proliferation and survival. Due to technical reasons such as freshness and instability of the material, these studies are extremely difficult in primary clinical samples. Therefore, we used bcr-abl transformed FDCP-mix p210^*bcr/abl*^ cells, a well characterised cell culture model system for CML (Pierce *et al*, 1998). These cells are conditionally transformed by expression of a temperature-sensitive p210^*bcr/abl*^ protein. They still remain IL-3 dependent although p210^*bcr/abl*^ sensitises them to the effects of IL-3, when cultured at the permissive temperature of 32°C. The main effect of p210^*bcr/abl*^ is to enhance viability under conditions of low IL-3 levels (0.01–0.1 ng ml^−1^) (Pierce *et al*, 1998).

**Figure 2:**
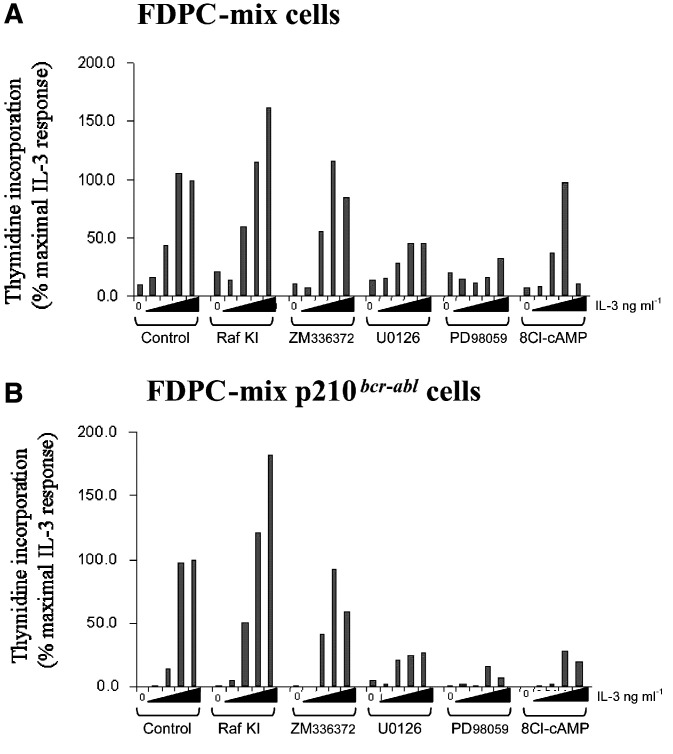
Effect of 8-Cl-cAMP, Raf kinase inhibitors and MEK inhibitors on the proliferation of FDCP-mix (**A**) and p210^*bcr-abl*^ transformed FDCP-mix cells (**B**). Cells were cultured at the permissive temperature in Fisher's medium with 20% horse serum and increasing concentrations of IL-3 (0, 0.01, 0.1, 1, 10 ng ml^−1^) as shown on the *X*-axis. 8-Cl-cAMP (100 *μ*M), MEK inhibitors U0126 (10 *μ*M) or PD98059 (50 *μ*M), and Raf kinase inhibitors, Raf kinase inhibitor I (Raf KI, 10 *μ*M) and ZM336372 (100 *μ*M) were added and DNA synthesis was assessed by measuring [3H] thymidine incorporation after 16 h. Experiments were carried out in triplicates.

Our previous studies with v-abl transformed fibroblasts (Weissinger *et al*, 1997) had indicated that PKA activation could downregulate the activity of Raf-1 and thereby cause apoptosis in these cells. This would provide a plausible explanation for the effects of 8-Cl-cAMP on CML cells. To test whether this hypothesis was also applicable to haematopoietic cells we compared 8-Cl-cAMP to selective pharmacological inhibitors of Raf-1 and MEK. As observed previously (Pierce *et al*, 1998) p210^*bcr/abl*^ did not significantly affect DNA synthesis when cells were compared to parental controls within 24 h after shifting them to the permissive temperature 32°C (Figure 2

). The two Raf kinase inhibitors (Raf KI and ZM336372) failed to interfere with IL-3 driven proliferation. Raf KI even accelerated proliferation in both the control cells and p210^*bcr/abl*^ cells exposed to 10 ng ml^−1^ IL-3. In contrast, both MEK inhibitors (U0126 and PD98059) interfered with DNA synthesis and this effect was slightly more pronounced in the p210^*bcr/abl*^ cells. In control cells 8-Cl-cAMP interfered with DNA synthesis only at high (10 ng ml^−1^) concentrations of IL-3, whereas it blocked proliferation in p210^*bcr/abl*^ cells at all concentrations.

**Figure 3:**
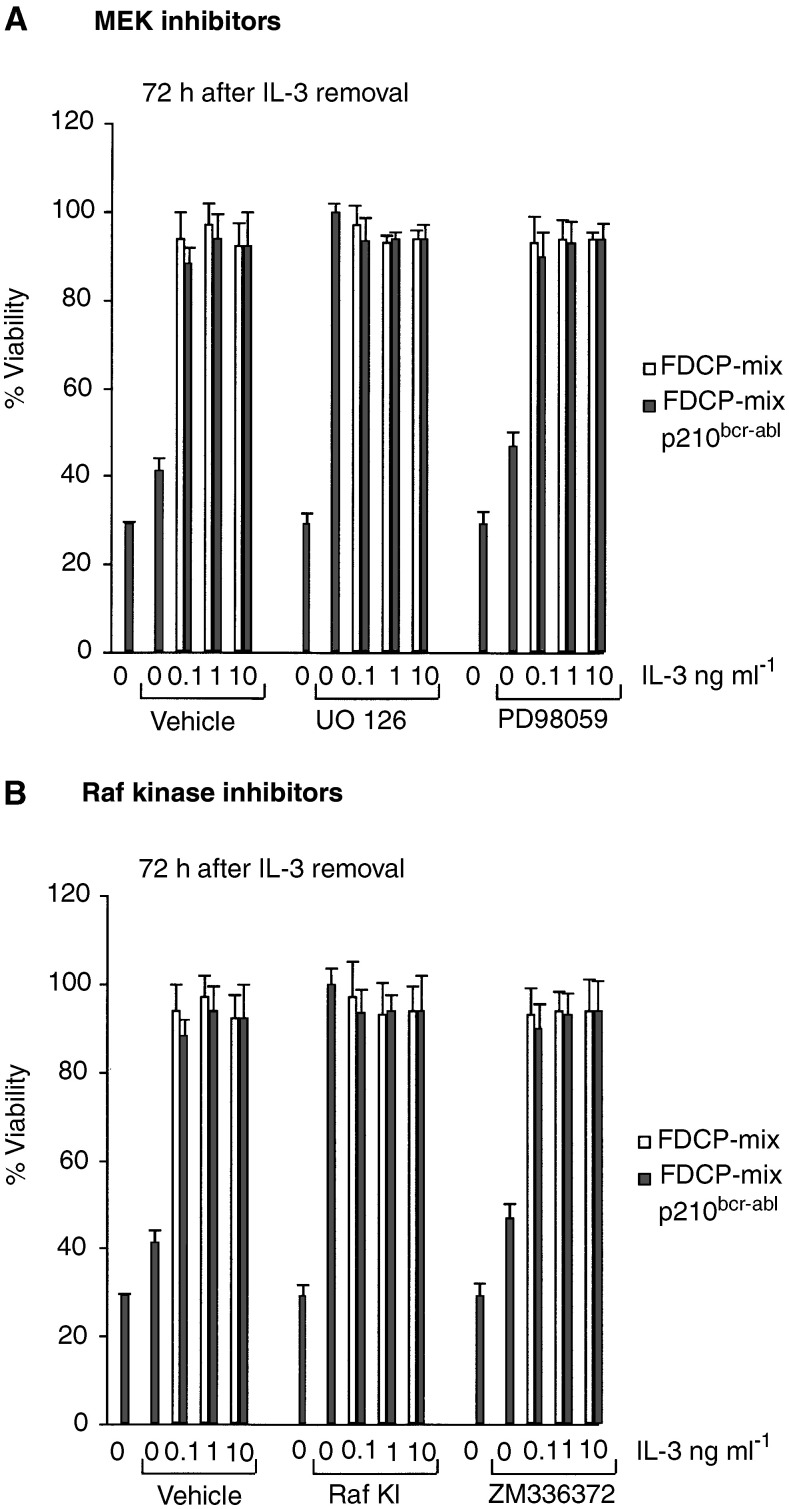
Effect of MEK inhibitors (**A**) and Raf kinase inhibitors (**B**) on the viability of FDCP-mix and p210^*bcr-abl*^ transformed FDCP-mix cells. Cells were cultured as in Figure 2. IL-3 was removed and inhibitors were added at the concentrations described in Figure 2. Cell viability was assessed by trypan blue exclusion 72 h after IL-3 removal. Experiments were carried out in triplicates.

A clear difference emerged when the effects on viability were assayed using trypan blue exclusion (Figure 3

). FDCP-mix cells have been reported to die by apoptosis following cytokine removal (Williams *et al*, 1990). p210^*bcr/abl*^ protected cells from the cytotoxic effects of IL-3 withdrawal, maintaining the viability of almost 40% of cells 3 days after IL-3 withdrawal. Under these conditions the viability of the control cells was severely compromised. IL-3 was a very potent survival factor even at 0.1 ng ml^−1^. Higher concentrations of IL-3 did not improve survival further. Interestingly, neither MEK inhibitors (Figure 3A) nor Raf inhibitors (Figure 3B) counteracted effects of p210^*bcr/abl*^ or IL-3 on cell viability.

**Figure 4:**
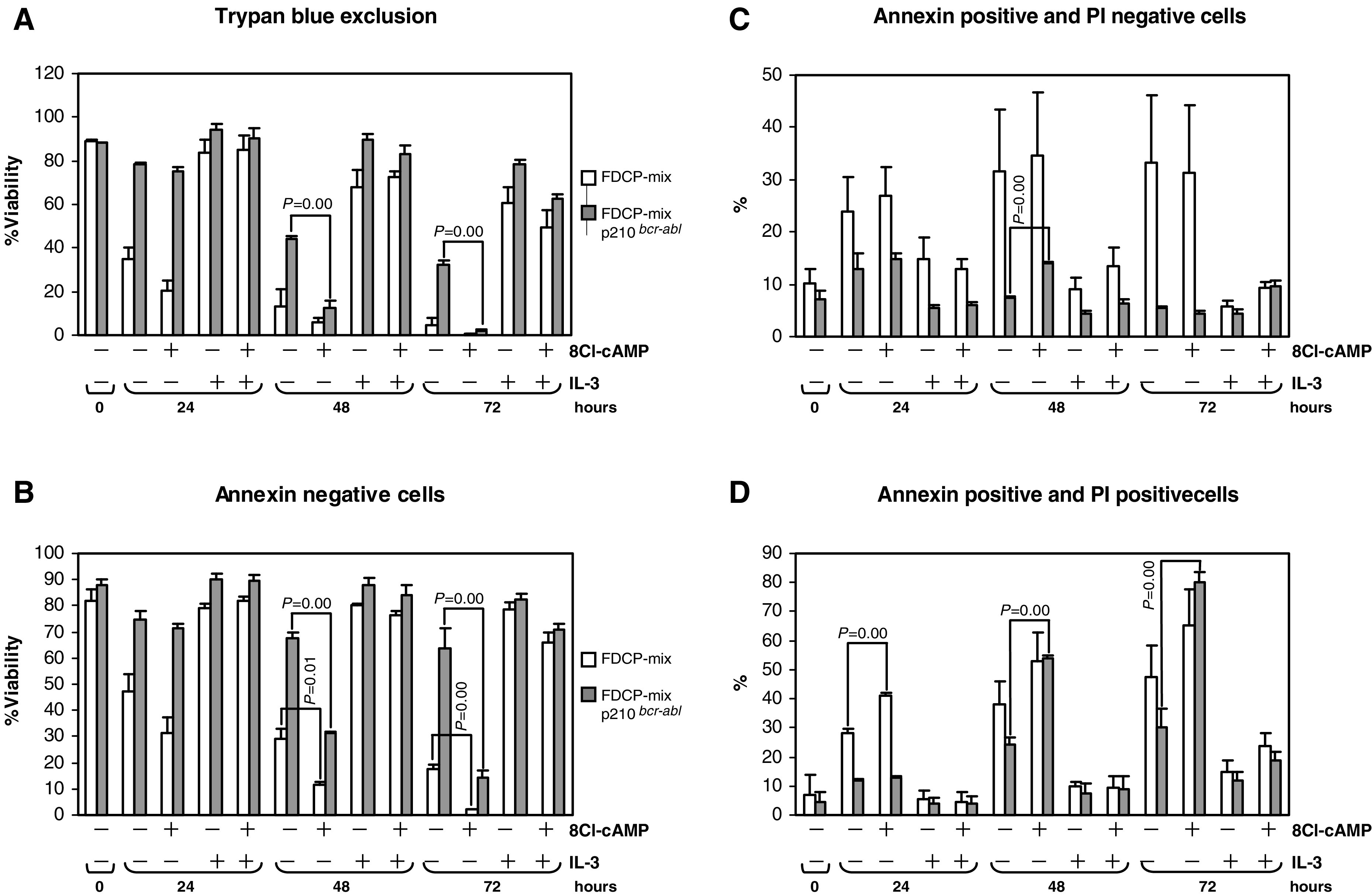
Analysis of the effect of 8-Cl-cAMP on the viability of FDCP-mix and p210^*bcr-abl*^ transformed FDCP-mix cells. Cells were cultured as in Figure 2. IL-3 was removed and 8-Cl-cAMP (100 *μ*M) was added. Cell viability was assessed 24, 48 and 72 h after IL-3 removal using (**A**) trypan blue exclusion, (**B**–**D**) Annexin and propidium iodide staining as described in the Materials and Methods section. Experiments were carried out in triplicates. The significance of changes was analysed by Student's paired *T*-test and significant changes are indicated in the figure along with the *P*-values.

In contrast, 8-Cl-cAMP significantly inhibited the cytoprotective effect of p210^*bcr/abl*^, but not of IL-3 (Figure 4A

). Moreover, 8-Cl-cAMP preferentially induced cell death in p210^*bcr/abl*^ as compared to control cells. This effect was most pronounced 48 and 72 h after IL-3 withdrawal, suggesting that p210^*bcr/abl*^ sensitises cells to killing by 8-Cl-cAMP. IL-3 protected against 8-Cl-cAMP induced cytotoxicity suggesting that IL-3 can activate p210^*bcr/abl*^ independent survival pathways. As trypan blue exclusion (Figure 4A) does not distinguish between necrotic and apoptotic cell death, we further tried to dissect the mode of 8-Cl-cAMP induced cell death. Apoptosis leads to cell surface phospholipid asymmetry resulting in the exposure of phosphatidylserine (PS) on the outer leaflet of the cytoplasmic membrane. Annexin V preferentially binds PS and has been used to detect apoptosis in the FDCP-mix cells (Francis *et al*, 2000). In contrast to necrosis, membrane integrity is maintained during apoptosis precluding staining of DNA by the membrane impermeable dye propidium iodide (PI). Thus, apoptosis is indicated by positive staining for annexin V and negative staining for PI. As shown in Figure 4B measuring viability as cells that escape apoptosis, that is, stain negative for annexin, largely parallels the data obtained with the trypan blue exclusion assay, exception that in this assay the parental FDCP-mix cells also show a significant decrease in viability in response to 8-Cl-cAMP 48 and 72 h after IL-3 removal. Measuring annexin positive and PI negative, that is, apoptotic cells (Figure 4C) showed a higher rate of apoptosis in the control cells under almost all conditions. 8-Cl-cAMP did not significantly affect this rate. However, a significant increase in apoptosis was observed in 8-Cl-cAMP treated p210^*bcr/abl*^ cells 48 h after IL-3 withdrawal. Necrotic cell death as measured by cells staining positive for annexin and PI (Figure 4D) was enhanced by 8-Cl-cAMP under conditions of IL-3 withdrawal. Significant increases were observed in control cell 24 h, and in the p210^*bcr/abl*^ cells 48 and 72 h after IL-3 removal. These results confirm the data obtained by the trypan blue exclusion assay, and suggest that 8-Cl-cAMP mediated cytotoxicity includes both apoptosis and necrotic cell death.

In summary, the inhibitor experiments demonstrated that the pathways mediating proliferation can be dissociated from pathways required for p210^*bcr/abl*^ driven cell survival. MEK–ERK signalling is required for DNA synthesis, but not for viability, whereas 8-Cl-cAMP can interfere with cell proliferation as well as survival. More importantly they show that 8-Cl-cAMP preferentially kills p210^*bcr/abl*^ cells.

## DISCUSSION

In this report we have analysed the influence of PKA-activation on transformed cells from eight patients with CML. The expression of p210^*bcr/abl*^ is a hallmark of CML. Among other signalling pathways p210^*bcr/abl*^ also activates the Raf–MEK–ERK pathway. We have previously shown that the inhibition of Raf-1 by 8-Cl-cAMP led to apoptosis in v-abl transformed fibroblasts, while control cells or cells expressing the v-raf oncogene showed only a reversible growth inhibition (Weissinger *et al*, 1997). Here we demonstrate that the activation of PKA is a promising approach to selectively eliminate the Ph1 chromosome positive progenitor cells from marrow obtained from CML-patients. Despite an initial reduction of total cell numbers no long-term cytotoxicity was observed when marrow cells from normal donors were treated with 50 or 100 *μ*M 8-CL-cAMP (Table 1). The initial decline in cell numbers is probably due to the inhibition of proliferation also observed in untransformed NIH3T3 fibroblasts or even in v-raf transformed fibroblasts (Weissinger *et al*, 1997). The reversible inhibition of normal cell proliferation could potentially be exploited to protect stem cells during chemotherapy. Thus, combining 8-Cl-cAMP with classical DNA damaging chemotherapeutic drugs may have the added benefits of assaulting the leukaemic cells by two routes while protecting the stem cells at the same time.

Seeking to understand the mechanism of growth inhibition and apoptosis induced in patient cells, we employed a well-characterised cell culture model of CML, that is, FDCP-mix cells expressing a temperature-sensitive p210^*bcr/abl*^ (Pierce *et al*, 1998). An obvious hypothesis emerging from our previous work with v-abl transformed fibroblasts (Weissinger *et al*, 1997) was that the inhibition of Raf-1, but not MEK, would be crucial for the cytotoxic effects of 8-Cl-cAMP. Therefore, we compared the effects of 8-Cl-cAMP to Raf-1 and MEK inhibitors. The results clearly show that MEK activity is required for the proliferation of both normal and p210^*bcr/abl*^ cells. In contrast, MEK activity was not required for p210^*bcr/abl*^ or IL-3 mediated viability. Curiously, Raf-1 inhibitors did not inhibit proliferation or survival, and Raf KI even enhanced these parameters. These results suggest that Raf-1 does not play a significant role in mediating proliferation or survival in these cells. However, the unexpected effects of Raf kinase inhibitors may be explained by a paradoxical activation of Raf previously observed with ZM 336372 (Hall-Jackson *et al*, 1999). Alternatively, Raf kinase activity may be dispensable for maintenance of cell viability, as Raf-1 has been shown to prevent apoptosis independent of its kinase activity by binding to and inhibiting the activity of the proapoptotic kinase ASK-1 (Chen *et al*, 2001). In this scenario, the Raf inhibitors would not be expected to show any effects, since they are ATP analogues that block kinase activity but not binding to other proteins. Furthermore, myeloid cells can activate the ERK pathway independent of Raf (Buscher *et al*, 1995), which could explain why the Raf and MEK inhibitors have different effects. Thus, the inhibitory effects of 8-Cl-cAMP on the proliferation and viability of p210^*bcr/abl*^ cells cannot be explained by the inhibition of the catalytic activities of Raf-1 and MEK.

Importantly, 8-Cl-cAMP exhibited significant selective cytotoxicity for cells that express p210^*bcr/abl*^. This was shown with the p210^*bcr/abl*^ transformed FDCP-mix cells as well as with primary bone marrow cells from leukaemic and normal donors. When marrow was obtained from patients in chronic phase of CML, a single incubation with 100 *μ*M 8-Cl-cAMP for 24 h was sufficient to completely eliminate the Ph1 chromosome positive progenitor cells *in vitro*. In analogy to our previously published experiments (Weissinger *et al*, 1997), the effects of 8-Cl-cAMP treatment and PKA activation were irreversible in p210^*bcr/abl*^ transformed progenitor cells of CML. Interestingly, in week 0 no elimination of the Ph1 chromosome positive progenitor cells had occurred. One explanation is that the induction of cell death requires proliferating cells. We reason that dividing cells are eliminated, whereas differentiating progenitors may not be affected by the activation of PKA. The induction of cell death in the susceptible progenitor population is reflected by the loss of Ph1 chromosome positive colonies after treatment as summarised in Table 2.

In conclusion, our results demonstrate that 8-Cl-cAMP can be useful for the efficient elimination of Ph1 chromosome positive progenitor cells from bone marrow *ex vivo* without severe toxic effects on normal cells. This offers a new method to purge marrow/stem cell populations from patients with Ph1 chromosome-positive leukaemias prior to autologous transplantation.
